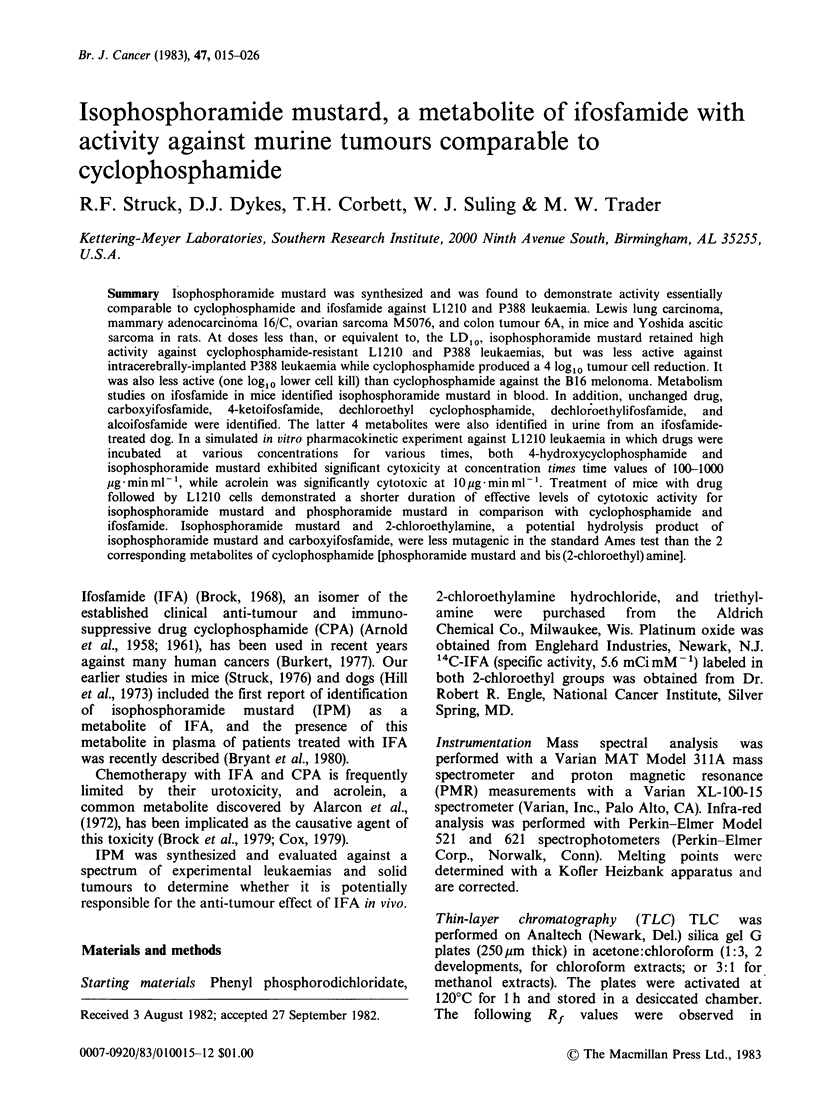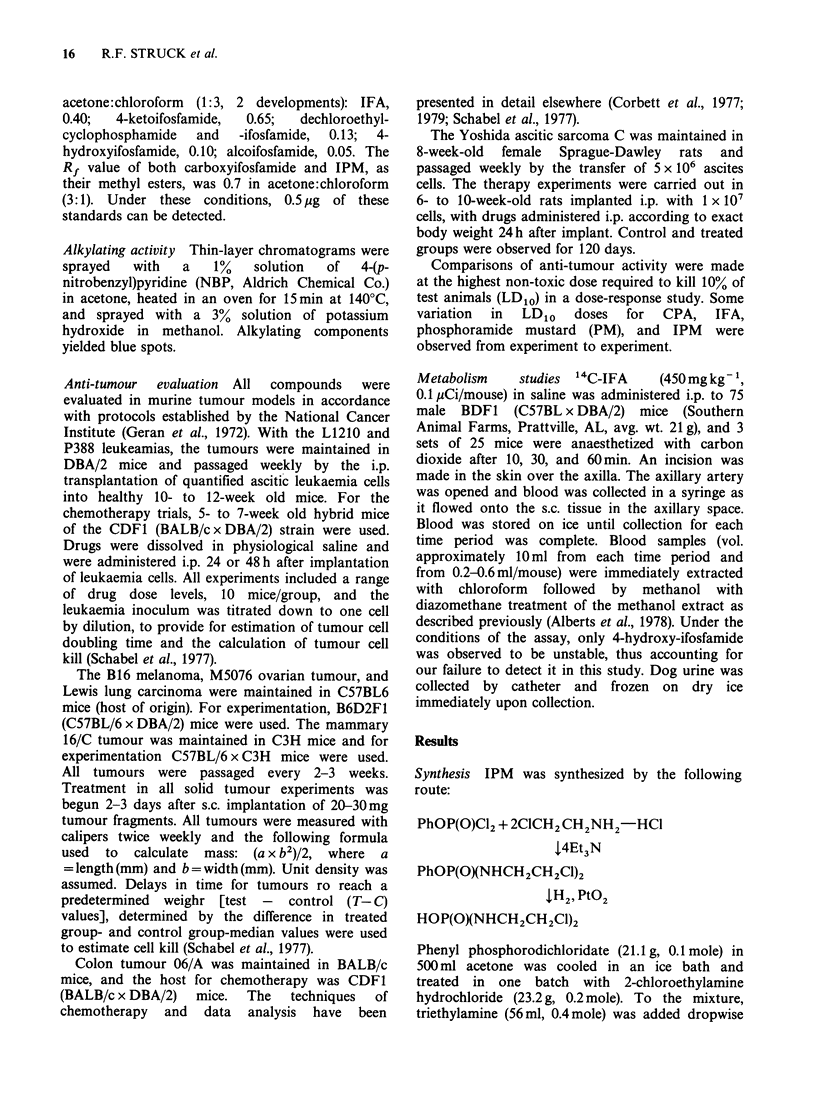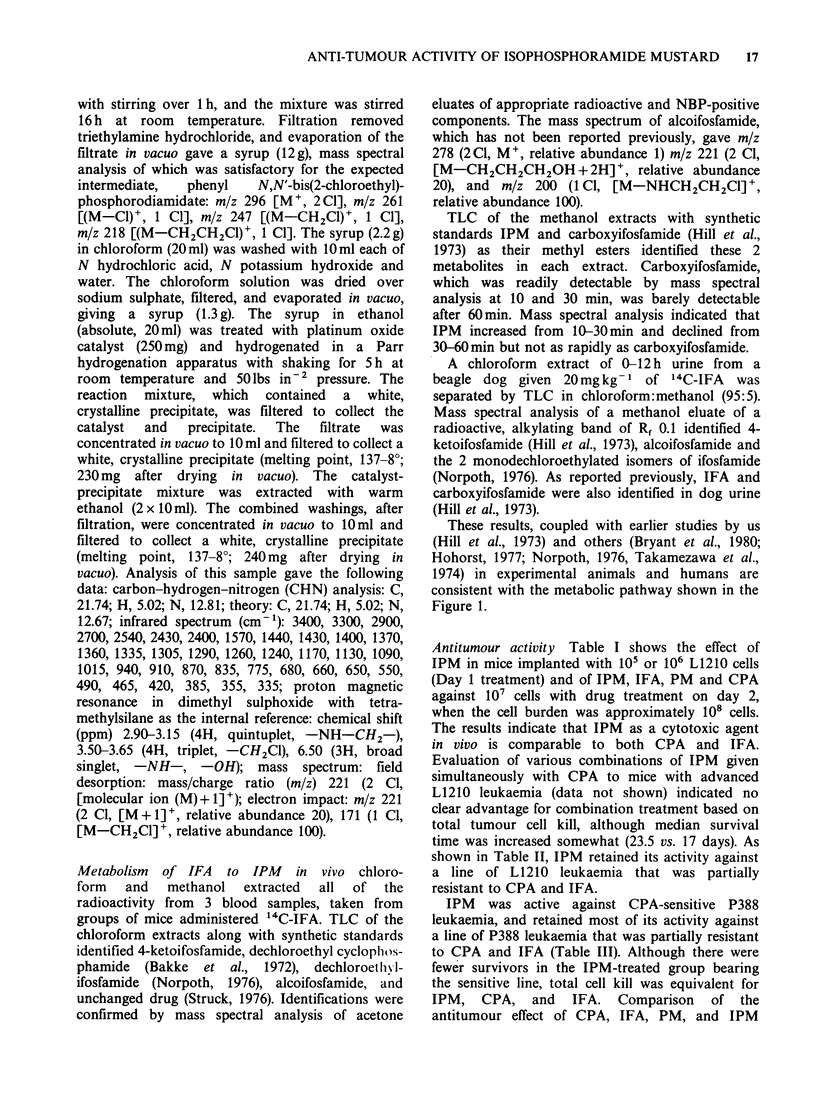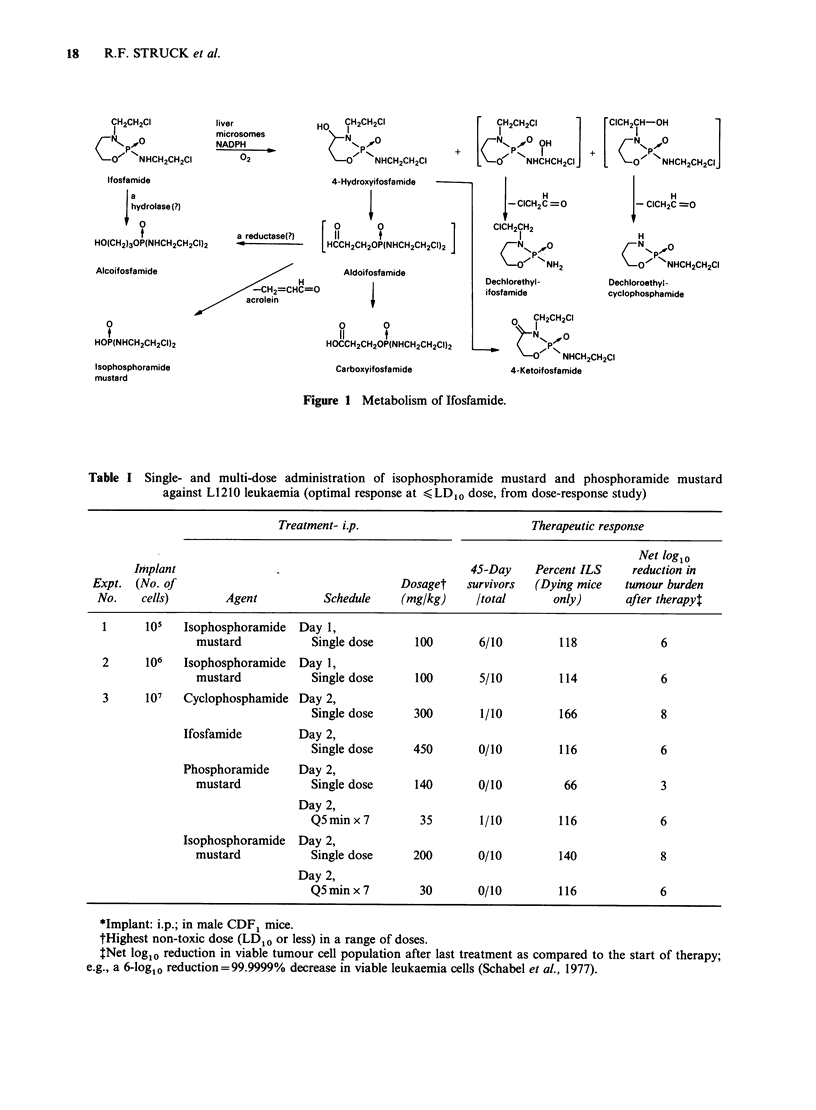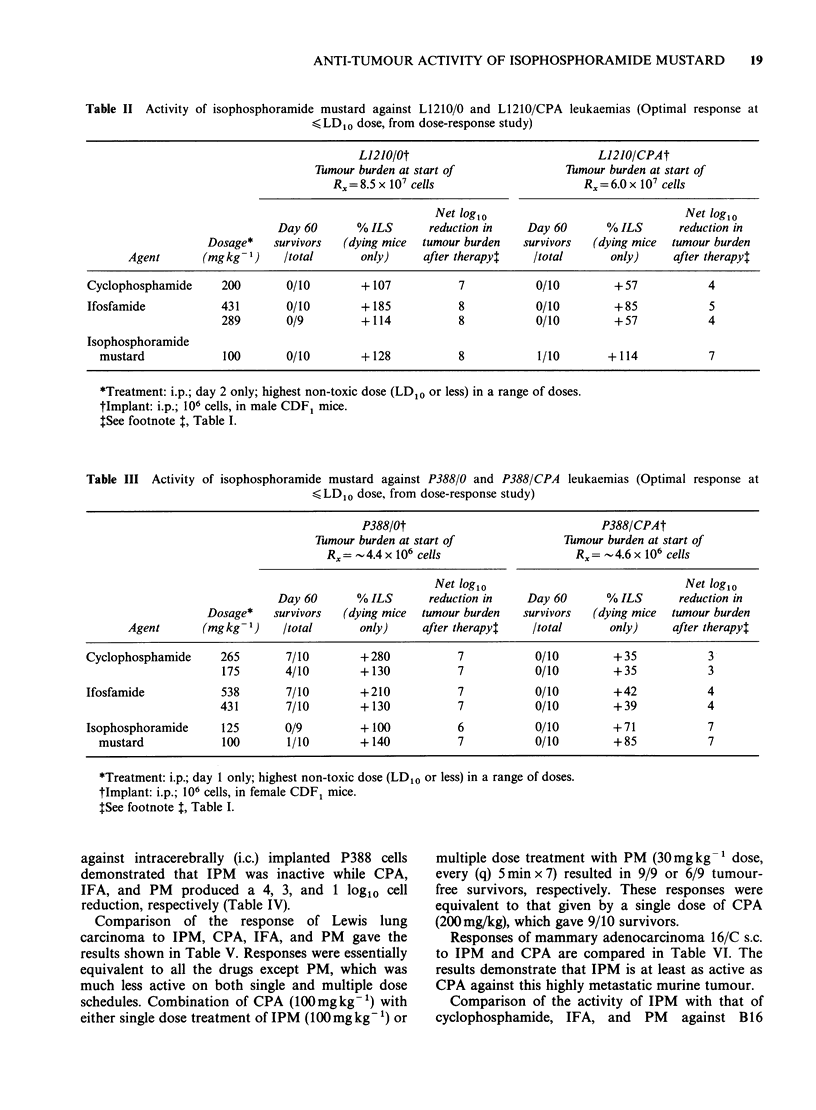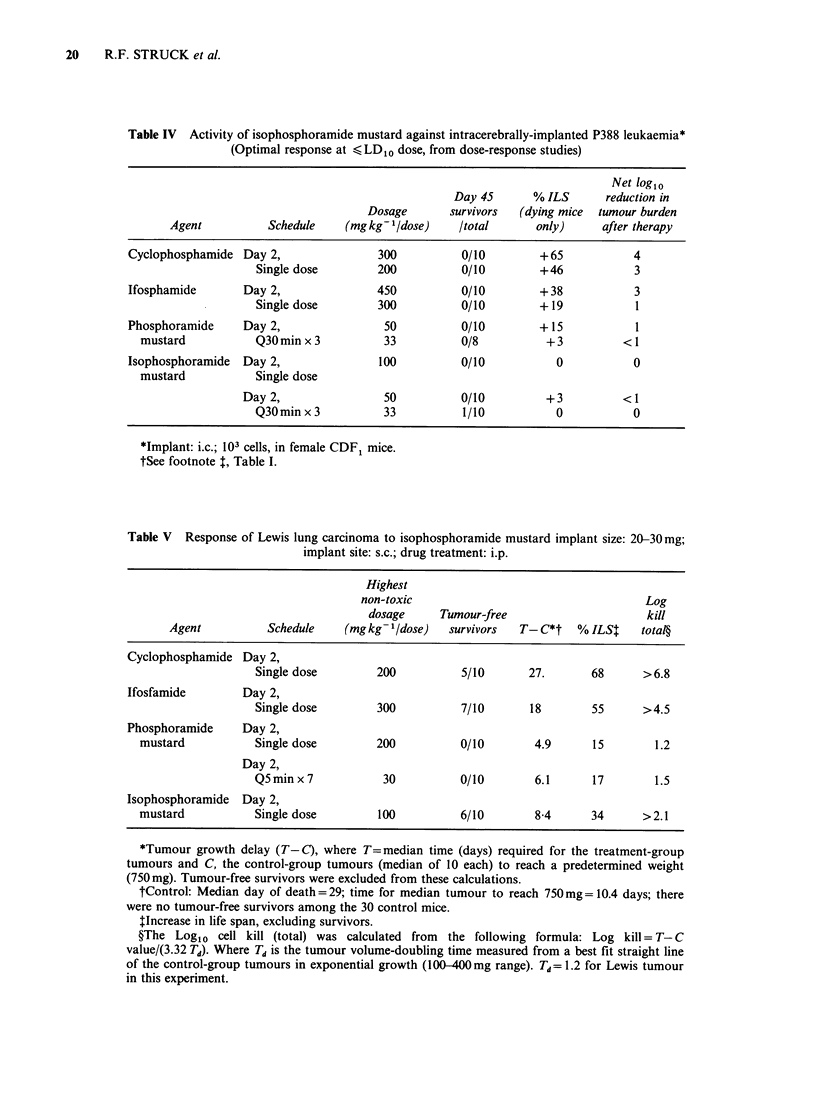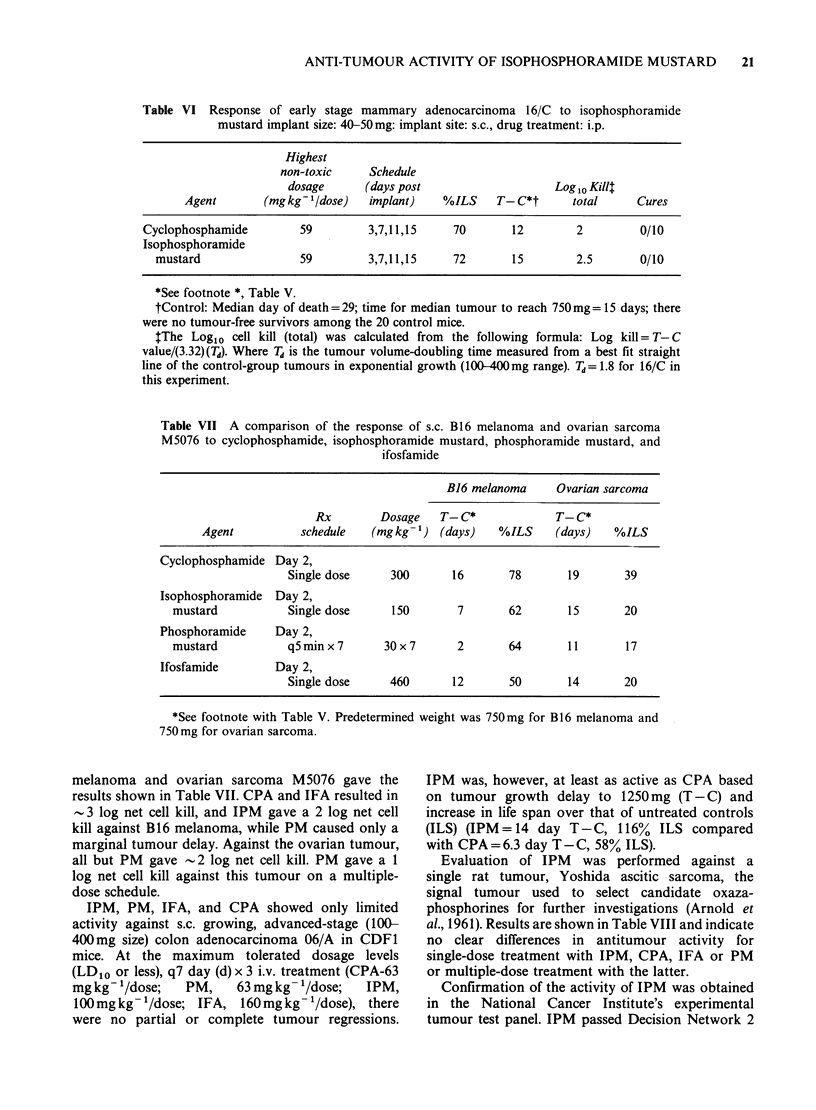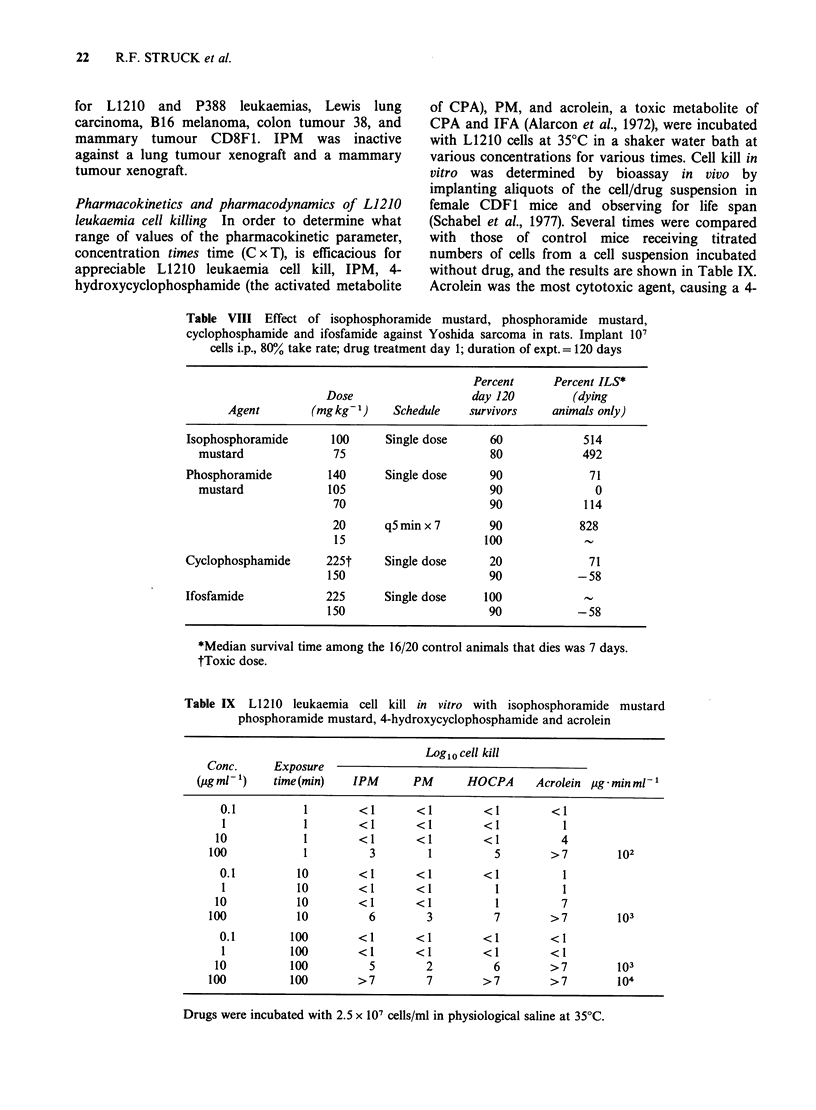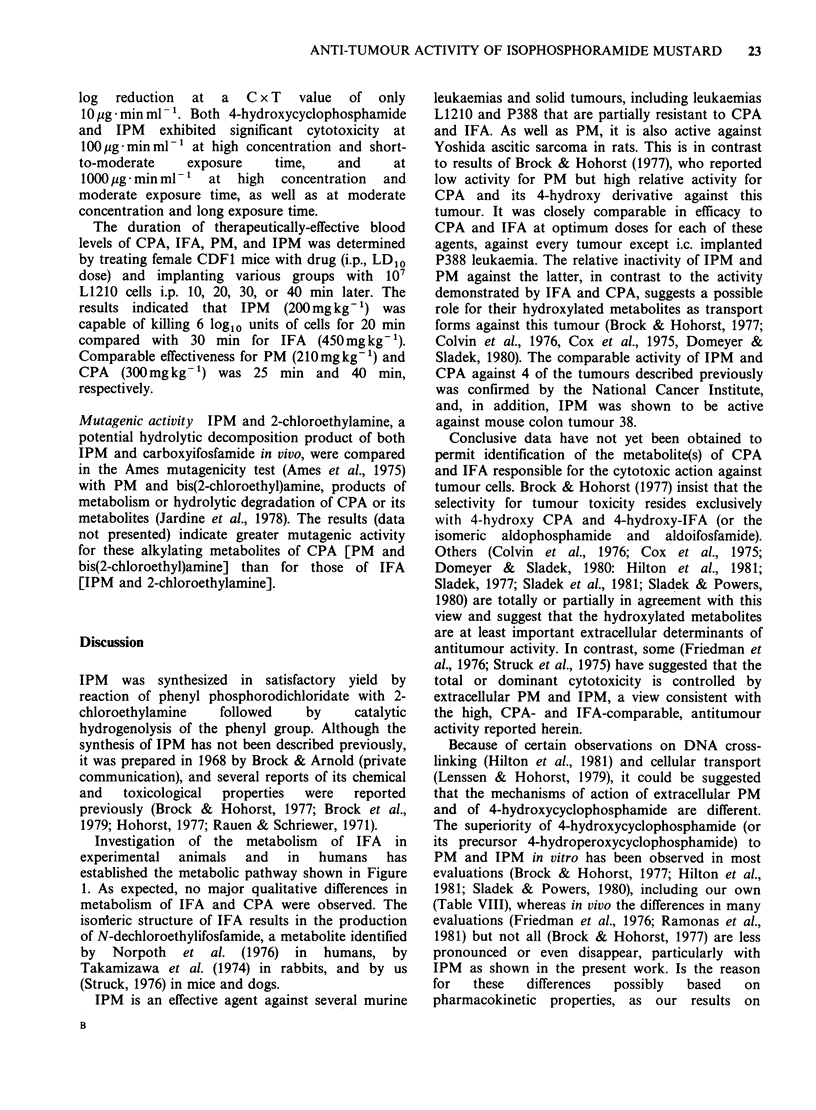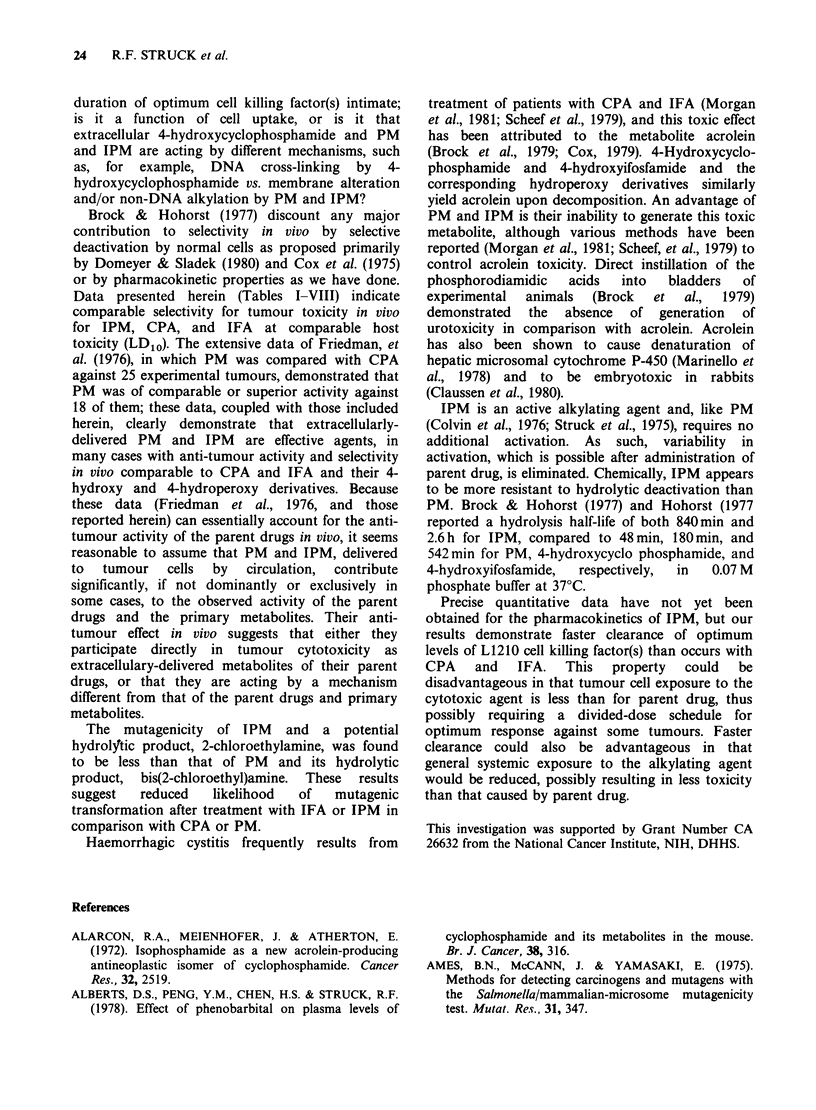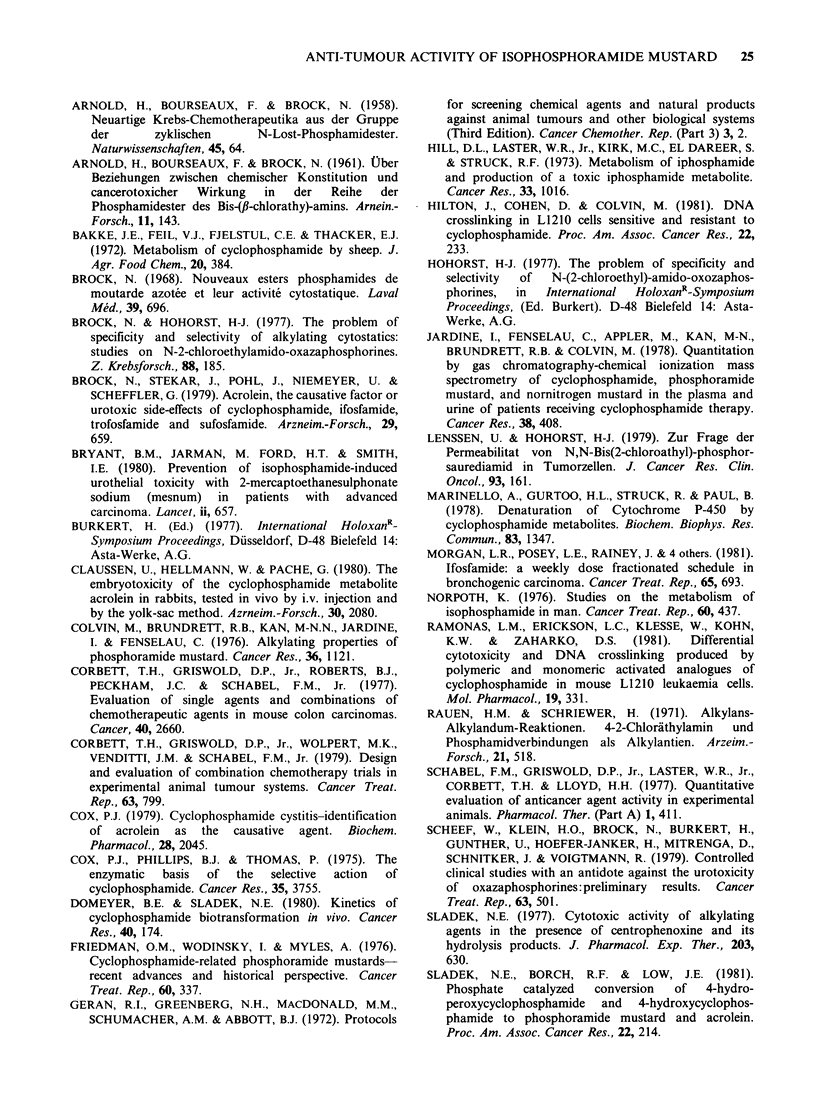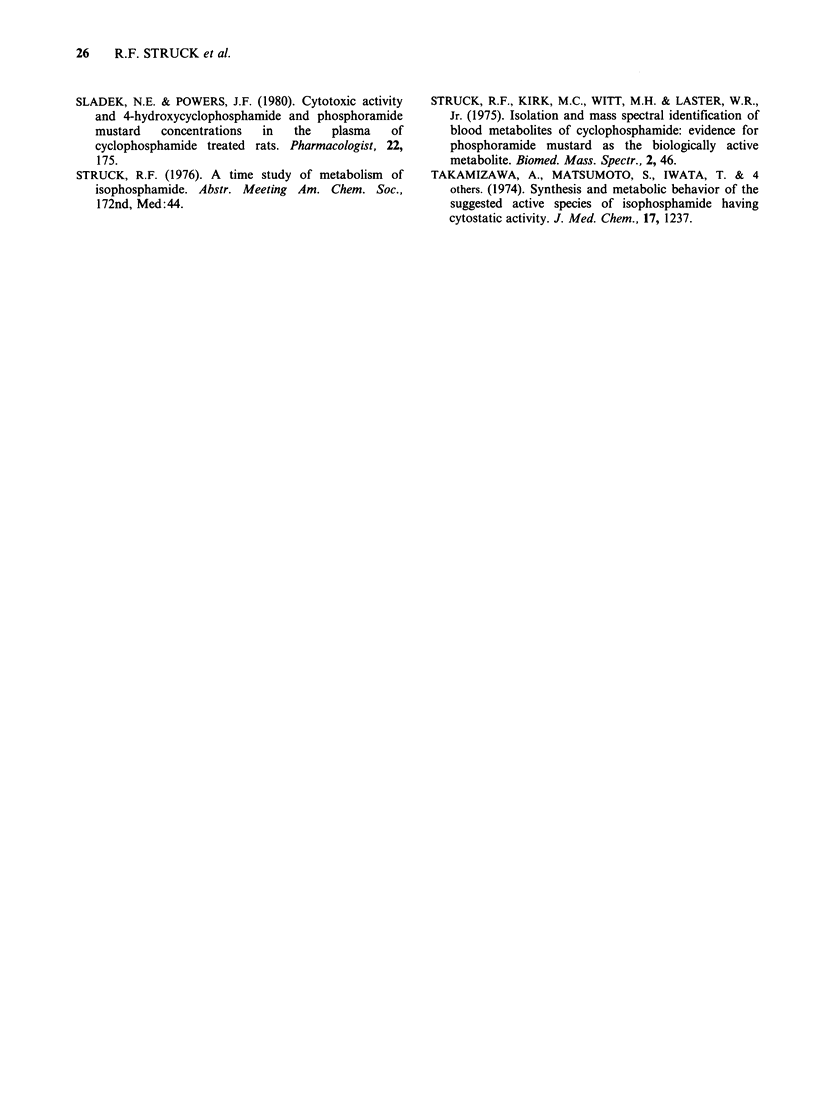# Isophosphoramide mustard, a metabolite of ifosfamide with activity against murine tumours comparable to cyclophosphamide.

**DOI:** 10.1038/bjc.1983.2

**Published:** 1983-01

**Authors:** R. F. Struck, D. J. Dykes, T. H. Corbett, W. J. Suling, M. W. Trader

## Abstract

Isophosphoramide mustard was synthesized and was found to demonstrate activity essentially comparable to cyclophosphamide and ifosfamide against L1210 and P388 leukaemia. Lewis lung carcinoma, mammary adenocarcinoma 16/C, ovarian sarcoma M5076, and colon tumour 6A, in mice and Yoshida ascitic sarcoma in rats. At doses less than, or equivalent to, the LD10, isophosphoramide mustard retained high activity against cyclophosphamide-resistant L1210 and P388 leukaemias, but was less active against intracerebrally-implanted P388 leukaemia while cyclophosphamide produced a 4 log10 tumour cell reduction. It was also less active (one log10 lower cell kill) than cyclophosphamide against the B16 melonoma. Metabolism studies on ifosfamide in mice identified isophosphoramide mustard in blood. In addition, unchanged drug, carboxyifosfamide, 4-ketoifosfamide, dechloroethyl cyclophosphamide, dechloroethylifosfamide, and alcoifosfamide were identified. The latter 4 metabolites were also identified in urine from an ifosfamide-treated dog. In a simulated in vitro pharmacokinetic experiment against L1210 leukaemia in which drugs were incubated at various concentrations for various times, both 4-hydroxycyclophosphamide and isophosphoramide mustard exhibited significant cytoxicity at concentration times time values of 100-1000 micrograms X min ml-1, while acrolein was significantly cytotoxic at 10 micrograms X min ml-1. Treatment of mice with drug followed by L1210 cells demonstrated a shorter duration of effective levels of cytotoxic activity for isophosphoramide mustard and phosphoramide mustard in comparison with cyclophosphamide and ifosfamide. Isophosphoramide mustard and 2-chloroethylamine, a potential hydrolysis product of isophosphoramide mustard and carboxyifosfamide, were less mutagenic in the standard Ames test than the 2 corresponding metabolites of cyclophosphamide [phosphoramide mustard and bis(2-chloroethyl)amine].


Br. J. Cancer (1983), **47**, 015–026

### Isophosphoramide mustard, a metabolite of ifosfamide with activity against murine tumours comparable to cyclophosphamide

R.F. Struck, D.J. Dykes, T.H. Corbett, W. J. Suling & M. W. Trader

*Kettering-Meyer Laboratories, Southern Research Institute, 2000 Ninth Avenue South, Birmingham, AL 35255, U.S.A.*

**Summary** Isophosphoramide mustard was synthesized and was found to demonstrate activity essentially comparable to cyclophosphamide and ifosfamide against L1210 and P388 leukaemia. Lewis lung carcinoma, mammary adenocarcinoma 16/C, ovarian sarcoma M5076, and colon tumour 6A, in mice and Yoshida ascitic sarcoma in rats. At doses less than, or equivalent to, the $LD_{10}$, isophosphoramide mustard retained high activity against cyclophosphamide-resistant L1210 and P388 leukaemias, but was less active against intracerebrally-implanted P388 leukaemia while cyclophosphamide produced a 4 $\log_{10}$ tumour cell reduction. It was also less active (one $\log_{10}$ lower cell kill) than cyclophosphamide against the B16 melonoma. Metabolism studies on ifosfamide in mice identified isophosphoramide mustard in blood. In addition, unchanged drug, carboxyifosfamide, 4-ketoifosfamide, dechloroethyl cyclophosphamide, dechloroethylifosfamide, and alcoifosfamide were identified. The latter 4 metabolites were also identified in urine from an ifosfamide-treated dog. In a simulated *in vitro* pharmacokinetic experiment against L1210 leukaemia in which drugs were incubated at various concentrations for various times, both 4-hydroxycyclophosphamide and isophosphoramide mustard exhibited significant cytoxicity at concentration *times* time values of 100–1000 $\mu g \cdot min\, ml^{-1}$, while acrolein was significantly cytotoxic at $10\,\mu g \cdot min\, ml^{-1}$. Treatment of mice with drug followed by L1210 cells demonstrated a shorter duration of effective levels of cytotoxic activity for isophosphoramide mustard and phosphoramide mustard in comparison with cyclophosphamide and ifosfamide. Isophosphoramide mustard and 2-chloroethylamine, a potential hydrolysis product of isophosphoramide mustard and carboxyifosfamide, were less mutagenic in the standard Ames test than the 2 corresponding metabolites of cyclophosphamide [phosphoramide mustard and bis(2-chloroethyl) amine].

Ifosfamide (IFA) (Brock, 1968), an isomer of the established clinical anti-tumour and immuno-suppressive drug cyclophosphamide (CPA) (Arnold *et al.*, 1958; 1961), has been used in recent years against many human cancers (Burkert, 1977). Our earlier studies in mice (Struck, 1976) and dogs (Hill *et al.*, 1973) included the first report of identification of isophosphoramide mustard (IPM) as a metabolite of IFA, and the presence of this metabolite in plasma of patients treated with IFA was recently described (Bryant *et al.*, 1980).

Chemotherapy with IFA and CPA is frequently limited by their urotoxicity, and acrolein, a common metabolite discovered by Alarcon *et al.*, (1972), has been implicated as the causative agent of this toxicity (Brock *et al.*, 1979; Cox, 1979).

IPM was synthesized and evaluated against a spectrum of experimental leukaemias and solid tumours to determine whether it is potentially responsible for the anti-tumour effect of IFA *in vivo*.

### Materials and methods

*Starting materials* Phenyl phosphorodichloridate, 2-chloroethylamine hydrochloride, and triethyl-amine were purchased from the Aldrich Chemical Co., Milwaukee, Wis. Platinum oxide was obtained from Englehard Industries, Newark, N.J. $^{14}C$-IFA (specific activity, 5.6 $mCi\,mM^{-1}$) labeled in both 2-chloroethyl groups was obtained from Dr. Robert R. Engle, National Cancer Institute, Silver Spring, MD.

*Instrumentation* Mass spectral analysis was performed with a Varian MAT Model 311A mass spectrometer and proton magnetic resonance (PMR) measurements with a Varian XL-100-15 spectrometer (Varian, Inc., Palo Alto, CA). Infra-red analysis was performed with Perkin-Elmer Model 521 and 621 spectrophotometers (Perkin–Elmer Corp., Norwalk, Conn). Melting points were determined with a Kofler Heizbank apparatus and are corrected.

*Thin-layer chromatography (TLC)* TLC was performed on Analtech (Newark, Del.) silica gel G plates (250 $\mu$m thick) in acetone:chloroform (1:3, 2 developments, for chloroform extracts; or 3:1 for methanol extracts). The plates were activated at 120°C for 1 h and stored in a desiccated chamber. The following $R_f$ values were observed in

Received 3 August 1982; accepted 27 September 1982.

0007-0920/83/010015–12 $01.00

© The Macmillan Press Ltd., 1983

acetone:chloroform (1:3, 2 developments): IFA, 0.40; 4-ketoifosfamide, 0.65; dechloroethylcyclophosphamide and -ifosfamide, 0.13; 4-hydroxyifosfamide, 0.10; alcoifosfamide, 0.05. The $R_f$ value of both carboxyifosfamide and IPM, as their methyl esters, was 0.7 in acetone:chloroform (3:1). Under these conditions, 0.5 $\mu$g of these standards can be detected.

*Alkylating activity* Thin-layer chromatograms were sprayed with a 1% solution of 4-(*p*-nitrobenzyl)pyridine (NBP, Aldrich Chemical Co.) in acetone, heated in an oven for 15 min at 140°C, and sprayed with a 3% solution of potassium hydroxide in methanol. Alkylating components yielded blue spots.

*Anti-tumour evaluation* All compounds were evaluated in murine tumour models in accordance with protocols established by the National Cancer Institute (Geran *et al.*, 1972). With the L1210 and P388 leukeamias, the tumours were maintained in DBA/2 mice and passaged weekly by the i.p. transplantation of quantified ascitic leukaemia cells into healthy 10- to 12-week old mice. For the chemotherapy trials, 5- to 7-week old hybrid mice of the CDF1 (BALB/c × DBA/2) strain were used. Drugs were dissolved in physiological saline and were administered i.p. 24 or 48 h after implantation of leukaemia cells. All experiments included a range of drug dose levels, 10 mice/group, and the leukaemia inoculum was titrated down to one cell by dilution, to provide for estimation of tumour cell doubling time and the calculation of tumour cell kill (Schabel *et al.*, 1977).

The B16 melanoma, M5076 ovarian tumour, and Lewis lung carcinoma were maintained in C57BL6 mice (host of origin). For experimentation, B6D2F1 (C57BL/6 × DBA/2) mice were used. The mammary 16/C tumour was maintained in C3H mice and for experimentation C57BL/6 × C3H mice were used. All tumours were passaged every 2–3 weeks. Treatment in all solid tumour experiments was begun 2–3 days after s.c. implantation of 20–30 mg tumour fragments. All tumours were measured with calipers twice weekly and the following formula used to calculate mass: $(a \times b^2)/2$, where $a$ = length (mm) and $b$ = width (mm). Unit density was assumed. Delays in time for tumours ro reach a predetermined weighr [test − control ($T-C$) values], determined by the difference in treated group- and control group-median values were used to estimate cell kill (Schabel *et al.*, 1977).

Colon tumour 06/A was maintained in BALB/c mice, and the host for chemotherapy was CDF1 (BALB/c × DBA/2) mice. The techniques of chemotherapy and data analysis have been presented in detail elsewhere (Corbett *et al.*, 1977; 1979; Schabel *et al.*, 1977).

The Yoshida ascitic sarcoma C was maintained in 8-week-old female Sprague-Dawley rats and passaged weekly by the transfer of $5 \times 10^6$ ascites cells. The therapy experiments were carried out in 6- to 10-week-old rats implanted i.p. with $1 \times 10^7$ cells, with drugs administered i.p. according to exact body weight 24 h after implant. Control and treated groups were observed for 120 days.

Comparisons of anti-tumour activity were made at the highest non-toxic dose required to kill 10% of test animals ($LD_{10}$) in a dose-response study. Some variation in $LD_{10}$ doses for CPA, IFA, phosphoramide mustard (PM), and IPM were observed from experiment to experiment.

*Metabolism studies* $^{14}$C-IFA (450 mg kg$^{-1}$, 0.1 $\mu$Ci/mouse) in saline was administered i.p. to 75 male BDF1 (C57BL × DBA/2) mice (Southern Animal Farms, Prattville, AL, avg. wt. 21 g), and 3 sets of 25 mice were anaesthetized with carbon dioxide after 10, 30, and 60 min. An incision was made in the skin over the axilla. The axillary artery was opened and blood was collected in a syringe as it flowed onto the s.c. tissue in the axillary space. Blood was stored on ice until collection for each time period was complete. Blood samples (vol. approximately 10 ml from each time period and from 0.2–0.6 ml/mouse) were immediately extracted with chloroform followed by methanol with diazomethane treatment of the methanol extract as described previously (Alberts *et al.*, 1978). Under the conditions of the assay, only 4-hydroxy-ifosfamide was observed to be unstable, thus accounting for our failure to detect it in this study. Dog urine was collected by catheter and frozen on dry ice immediately upon collection.

### Results

*Synthesis* IPM was synthesized by the following route:

$$PhOP(O)Cl_2 + 2ClCH_2CH_2NH_2\text{—}HCl$$

$$\downarrow 4Et_3N$$

$$PhOP(O)(NHCH_2CH_2Cl)_2$$

$$\downarrow H_2, PtO_2$$

$$HOP(O)(NHCH_2CH_2Cl)_2$$

Phenyl phosphorodichloridate (21.1 g, 0.1 mole) in 500 ml acetone was cooled in an ice bath and treated in one batch with 2-chloroethylamine hydrochloride (23.2 g, 0.2 mole). To the mixture, triethylamine (56 ml, 0.4 mole) was added dropwise with stirring over 1 h, and the mixture was stirred 16 h at room temperature. Filtration removed triethylamine hydrochloride, and evaporation of the filtrate *in vacuo* gave a syrup (12 g), mass spectral analysis of which was satisfactory for the expected intermediate, phenyl *N,N'*-bis(2-chloroethyl)-phosphorodiamidate: *m/z* 296 [$M^+$, 2 Cl], *m/z* 261 [$(M—Cl)^+$, 1 Cl], *m/z* 247 [$(M—CH_2Cl)^+$, 1 Cl], *m/z* 218 [$(M—CH_2CH_2Cl)^+$, 1 Cl]. The syrup (2.2 g) in chloroform (20 ml) was washed with 10 ml each of *N* hydrochloric acid, *N* potassium hydroxide and water. The chloroform solution was dried over sodium sulphate, filtered, and evaporated *in vacuo*, giving a syrup (1.3 g). The syrup in ethanol (absolute, 20 ml) was treated with platinum oxide catalyst (250 mg) and hydrogenated in a Parr hydrogenation apparatus with shaking for 5 h at room temperature and 50 lbs $in^{-2}$ pressure. The reaction mixture, which contained a white, crystalline precipitate, was filtered to collect the catalyst and precipitate. The filtrate was concentrated *in vacuo* to 10 ml and filtered to collect a white, crystalline precipitate (melting point, 137–8°; 230 mg after drying *in vacuo*). The catalyst-precipitate mixture was extracted with warm ethanol (2 x 10 ml). The combined washings, after filtration, were concentrated *in vacuo* to 10 ml and filtered to collect a white, crystalline precipitate (melting point, 137–8°; 240 mg after drying *in vacuo*). Analysis of this sample gave the following data: carbon–hydrogen–nitrogen (CHN) analysis: C, 21.74; H, 5.02; N, 12.81; theory: C, 21.74; H, 5.02; N, 12.67; infrared spectrum ($cm^{-1}$): 3400, 3300, 2900, 2700, 2540, 2430, 2400, 1570, 1440, 1430, 1400, 1370, 1360, 1335, 1305, 1290, 1260, 1240, 1170, 1130, 1090, 1015, 940, 910, 870, 835, 775, 680, 660, 650, 550, 490, 465, 420, 385, 355, 335; proton magnetic resonance in dimethyl sulphoxide with tetra-methylsilane as the internal reference: chemical shift (ppm) 2.90–3.15 (4H, quintuplet, $—NH—CH_2—$), 3.50–3.65 (4H, triplet, $—CH_2Cl$), 6.50 (3H, broad singlet, *—NH—*, *—OH*); mass spectrum: field desorption: mass/charge ratio (*m/z*) 221 (2 Cl, [molecular ion $(M)+1]^+$); electron impact: *m/z* 221 (2 Cl, $[M+1]^+$, relative abundance 20), 171 (1 Cl, $[M—CH_2Cl]^+$, relative abundance 100).

*Metabolism of IFA to IPM in vivo* chloroform and methanol extracted all of the radioactivity from 3 blood samples, taken from groups of mice administered $^{14}$C-IFA. TLC of the chloroform extracts along with synthetic standards identified 4-ketoifosfamide, dechloroethyl cyclophosphamide (Bakke *et al.*, 1972), dechloroethyl-ifosfamide (Norpoth, 1976), alcoifosfamide, and unchanged drug (Struck, 1976). Identifications were confirmed by mass spectral analysis of acetone eluates of appropriate radioactive and NBP-positive components. The mass spectrum of alcoifosfamide, which has not been reported previously, gave *m/z* 278 (2 Cl, $M^+$, relative abundance 1) *m/z* 221 (2 Cl, $[M—CH_2CH_2CH_2OH+2H]^+$, relative abundance 20), and *m/z* 200 (1 Cl, $[M—NHCH_2CH_2Cl]^+$, relative abundance 100).

TLC of the methanol extracts with synthetic standards IPM and carboxyifosfamide (Hill *et al.*, 1973) as their methyl esters identified these 2 metabolites in each extract. Carboxyifosfamide, which was readily detectable by mass spectral analysis at 10 and 30 min, was barely detectable after 60 min. Mass spectral analysis indicated that IPM increased from 10–30 min and declined from 30–60 min but not as rapidly as carboxyifosfamide.

A chloroform extract of 0–12 h urine from a beagle dog given 20 mg $kg^{-1}$ of $^{14}$C-IFA was separated by TLC in chloroform:methanol (95:5). Mass spectral analysis of a methanol eluate of a radioactive, alkylating band of $R_f$ 0.1 identified 4-ketoifosfamide (Hill *et al.*, 1973), alcoifosfamide and the 2 monodechloroethylated isomers of ifosfamide (Norpoth, 1976). As reported previously, IFA and carboxyifosfamide were also identified in dog urine (Hill *et al.*, 1973).

These results, coupled with earlier studies by us (Hill *et al.*, 1973) and others (Bryant *et al.*, 1980; Hohorst, 1977; Norpoth, 1976, Takamezawa *et al.*, 1974) in experimental animals and humans are consistent with the metabolic pathway shown in the Figure 1.

*Antitumour activity* Table I shows the effect of IPM in mice implanted with $10^5$ or $10^6$ L1210 cells (Day 1 treatment) and of IPM, IFA, PM and CPA against $10^7$ cells with drug treatment on day 2, when the cell burden was approximately $10^8$ cells. The results indicate that IPM as a cytotoxic agent *in vivo* is comparable to both CPA and IFA. Evaluation of various combinations of IPM given simultaneously with CPA to mice with advanced L1210 leukaemia (data not shown) indicated no clear advantage for combination treatment based on total tumour cell kill, although median survival time was increased somewhat (23.5 *vs.* 17 days). As shown in Table II, IPM retained its activity against a line of L1210 leukaemia that was partially resistant to CPA and IFA.

IPM was active against CPA-sensitive P388 leukaemia, and retained most of its activity against a line of P388 leukaemia that was partially resistant to CPA and IFA (Table III). Although there were fewer survivors in the IPM-treated group bearing the sensitive line, total cell kill was equivalent for IPM, CPA, and IFA. Comparison of the antitumour effect of CPA, IFA, PM, and IPM

**Figure 1** Metabolism of Ifosfamide.

**Table I** Single- and multi-dose administration of isophosphoramide mustard and phosphoramide mustard against L1210 leukaemia (optimal response at $\leqslant LD_{10}$ dose, from dose-response study)

| | | Treatment- i.p. | | | Therapeutic response | | |
|---|---|---|---|---|---|---|---|
| *Expt. No.* | *Implant (No. of cells)* | *Agent* | *Schedule* | *Dosage† (mg/kg)* | *45-Day survivors /total* | *Percent ILS (Dying mice only)* | *Net $log_{10}$ reduction in tumour burden after therapy‡* |
| 1 | $10^5$ | Isophosphoramide mustard | Day 1, Single dose | 100 | 6/10 | 118 | 6 |
| 2 | $10^6$ | Isophosphoramide mustard | Day 1, Single dose | 100 | 5/10 | 114 | 6 |
| 3 | $10^7$ | Cyclophosphamide | Day 2, Single dose | 300 | 1/10 | 166 | 8 |
| | | Ifosfamide | Day 2, Single dose | 450 | 0/10 | 116 | 6 |
| | | Phosphoramide mustard | Day 2, Single dose | 140 | 0/10 | 66 | 3 |
| | | | Day 2, Q5 min x 7 | 35 | 1/10 | 116 | 6 |
| | | Isophosphoramide mustard | Day 2, Single dose | 200 | 0/10 | 140 | 8 |
| | | | Day 2, Q5 min x 7 | 30 | 0/10 | 116 | 6 |

*Implant: i.p.; in male $CDF_1$ mice.

†Highest non-toxic dose ($LD_{10}$ or less) in a range of doses.

‡Net $log_{10}$ reduction in viable tumour cell population after last treatment as compared to the start of therapy; e.g., a 6-$log_{10}$ reduction = 99.9999% decrease in viable leukaemia cells (Schabel *et al.*, 1977).

**Table II** Activity of isophosphoramide mustard against L1210/0 and L1210/CPA leukaemias (Optimal response at $\leqslant LD_{10}$ dose, from dose-response study)

| | | *L1210/0*† *Tumour burden at start of* $R_x = 8.5 \times 10^7$ *cells* | | | *L1210/CPA*† *Tumour burden at start of* $R_x = 6.0 \times 10^7$ *cells* | | |
|---|---|---|---|---|---|---|---|
| *Agent* | *Dosage** *(mg kg⁻¹)* | *Day 60 survivors /total* | *% ILS (dying mice only)* | *Net* $log_{10}$ *reduction in tumour burden after therapy*‡ | *Day 60 survivors /total* | *% ILS (dying mice only)* | *Net* $log_{10}$ *reduction in tumour burden after therapy*‡ |
| Cyclophosphamide | 200 | 0/10 | +107 | 7 | 0/10 | +57 | 4 |
| Ifosfamide | 431 | 0/10 | +185 | 8 | 0/10 | +85 | 5 |
| | 289 | 0/9 | +114 | 8 | 0/10 | +57 | 4 |
| Isophosphoramide mustard | 100 | 0/10 | +128 | 8 | 1/10 | +114 | 7 |

*Treatment: i.p.; day 2 only; highest non-toxic dose ($LD_{10}$ or less) in a range of doses.
†Implant: i.p.; $10^6$ cells, in male $CDF_1$ mice.
‡See footnote ‡, Table I.

**Table III** Activity of isophosphoramide mustard against *P388/0* and *P388/CPA* leukaemias (Optimal response at $\leqslant LD_{10}$ dose, from dose-response study)

| | | *P388/0*† *Tumour burden at start of* $R_x = \sim 4.4 \times 10^6$ *cells* | | | *P388/CPA*† *Tumour burden at start of* $R_x = \sim 4.6 \times 10^6$ *cells* | | |
|---|---|---|---|---|---|---|---|
| *Agent* | *Dosage** *(mg kg⁻¹)* | *Day 60 survivors /total* | *% ILS (dying mice only)* | *Net* $log_{10}$ *reduction in tumour burden after therapy*‡ | *Day 60 survivors /total* | *% ILS (dying mice only)* | *Net* $log_{10}$ *reduction in tumour burden after therapy*‡ |
| Cyclophosphamide | 265 | 7/10 | +280 | 7 | 0/10 | +35 | 3 |
| | 175 | 4/10 | +130 | 7 | 0/10 | +35 | 3 |
| Ifosfamide | 538 | 7/10 | +210 | 7 | 0/10 | +42 | 4 |
| | 431 | 7/10 | +130 | 7 | 0/10 | +39 | 4 |
| Isophosphoramide | 125 | 0/9 | +100 | 6 | 0/10 | +71 | 7 |
| mustard | 100 | 1/10 | +140 | 7 | 0/10 | +85 | 7 |

*Treatment: i.p.; day 1 only; highest non-toxic dose ($LD_{10}$ or less) in a range of doses.
†Implant: i.p.; $10^6$ cells, in female $CDF_1$ mice.
‡See footnote ‡, Table I.

against intracerebrally (i.c.) implanted P388 cells demonstrated that IPM was inactive while CPA, IFA, and PM produced a 4, 3, and 1 $\log_{10}$ cell reduction, respectively (Table IV).

Comparison of the response of Lewis lung carcinoma to IPM, CPA, IFA, and PM gave the results shown in Table V. Responses were essentially equivalent to all the drugs except PM, which was much less active on both single and multiple dose schedules. Combination of CPA (100 mg kg$^{-1}$) with either single dose treatment of IPM (100 mg kg$^{-1}$) or multiple dose treatment with PM (30 mg kg$^{-1}$ dose, every (q) 5 min × 7) resulted in 9/9 or 6/9 tumour-free survivors, respectively. These responses were equivalent to that given by a single dose of CPA (200 mg/kg), which gave 9/10 survivors.

Responses of mammary adenocarcinoma 16/C s.c. to IPM and CPA are compared in Table VI. The results demonstrate that IPM is at least as active as CPA against this highly metastatic murine tumour.

Comparison of the activity of IPM with that of cyclophosphamide, IFA, and PM against B16

**Table IV** Activity of isophosphoramide mustard against intracerebrally-implanted P388 leukaemia* (Optimal response at $\leqslant LD_{10}$ dose, from dose-response studies)

| *Agent* | *Schedule* | *Dosage (mg kg$^{-1}$/dose)* | *Day 45 survivors /total* | *% ILS (dying mice only)* | *Net $\log_{10}$ reduction in tumour burden after therapy* |
|---|---|---|---|---|---|
| Cyclophosphamide | Day 2, | 300 | 0/10 | +65 | 4 |
| | Single dose | 200 | 0/10 | +46 | 3 |
| Ifosphamide | Day 2, | 450 | 0/10 | +38 | 3 |
| | Single dose | 300 | 0/10 | +19 | 1 |
| Phosphoramide | Day 2, | 50 | 0/10 | +15 | 1 |
| mustard | Q30 min x 3 | 33 | 0/8 | +3 | <1 |
| Isophosphoramide | Day 2, | 100 | 0/10 | 0 | 0 |
| mustard | Single dose | | | | |
| | Day 2, | 50 | 0/10 | +3 | <1 |
| | Q30 min x 3 | 33 | 1/10 | 0 | 0 |

*Implant: i.c.; $10^3$ cells, in female $CDF_1$ mice.
†See footnote ‡, Table I.

**Table V** Response of Lewis lung carcinoma to isophosphoramide mustard implant size: 20–30 mg; implant site: s.c.; drug treatment: i.p.

| *Agent* | *Schedule* | *Highest non-toxic dosage (mg kg$^{-1}$/dose)* | *Tumour-free survivors* | *T−C*†* | *% ILS‡* | *Log kill total§* |
|---|---|---|---|---|---|---|
| Cyclophosphamide | Day 2, Single dose | 200 | 5/10 | 27. | 68 | >6.8 |
| Ifosfamide | Day 2, Single dose | 300 | 7/10 | 18 | 55 | >4.5 |
| Phosphoramide mustard | Day 2, Single dose | 200 | 0/10 | 4.9 | 15 | 1.2 |
| | Day 2, Q5 min x 7 | 30 | 0/10 | 6.1 | 17 | 1.5 |
| Isophosphoramide mustard | Day 2, Single dose | 100 | 6/10 | 8·4 | 34 | >2.1 |

*Tumour growth delay ($T-C$), where $T$ = median time (days) required for the treatment-group tumours and $C$, the control-group tumours (median of 10 each) to reach a predetermined weight (750 mg). Tumour-free survivors were excluded from these calculations.

†Control: Median day of death = 29; time for median tumour to reach 750 mg = 10.4 days; there were no tumour-free survivors among the 30 control mice.

‡Increase in life span, excluding survivors.

§The $\text{Log}_{10}$ cell kill (total) was calculated from the following formula: Log kill = $T-C$ value/(3.32 $T_d$). Where $T_d$ is the tumour volume-doubling time measured from a best fit straight line of the control-group tumours in exponential growth (100–400 mg range). $T_d$ = 1.2 for Lewis tumour in this experiment.

**Table VI** Response of early stage mammary adenocarcinoma 16/C to isophosphoramide mustard implant size: 40–50 mg: implant site: s.c., drug treatment: i.p.

| *Agent* | *Highest non-toxic dosage (mg kg$^{-1}$/dose)* | *Schedule (days post implant)* | *%ILS* | *T−C*†* | *Log$_{10}$ Kill‡ total* | *Cures* |
|---|---|---|---|---|---|---|
| Cyclophosphamide | 59 | 3,7,11,15 | 70 | 12 | 2 | 0/10 |
| Isophosphoramide mustard | 59 | 3,7,11,15 | 72 | 15 | 2.5 | 0/10 |

*See footnote *, Table V.

†Control: Median day of death = 29; time for median tumour to reach 750 mg = 15 days; there were no tumour-free survivors among the 20 control mice.

‡The $\text{Log}_{10}$ cell kill (total) was calculated from the following formula: Log kill = $T-C$ value/(3.32)($T_d$). Where $T_d$ is the tumour volume-doubling time measured from a best fit straight line of the control-group tumours in exponential growth (100–400 mg range). $T_d = 1.8$ for 16/C in this experiment.

**Table VII** A comparison of the response of s.c. B16 melanoma and ovarian sarcoma M5076 to cyclophosphamide, isophosphoramide mustard, phosphoramide mustard, and ifosfamide

| | | | *B16 melanoma* | | *Ovarian sarcoma* | |
|---|---|---|---|---|---|---|
| *Agent* | *Rx schedule* | *Dosage (mg kg$^{-1}$)* | *T−C* (days)* | *%ILS* | *T−C* (days)* | *%ILS* |
| Cyclophosphamide | Day 2, Single dose | 300 | 16 | 78 | 19 | 39 |
| Isophosphoramide mustard | Day 2, Single dose | 150 | 7 | 62 | 15 | 20 |
| Phosphoramide mustard | Day 2, q5 min × 7 | 30 × 7 | 2 | 64 | 11 | 17 |
| Ifosfamide | Day 2, Single dose | 460 | 12 | 50 | 14 | 20 |

*See footnote with Table V. Predetermined weight was 750 mg for B16 melanoma and 750 mg for ovarian sarcoma.

melanoma and ovarian sarcoma M5076 gave the results shown in Table VII. CPA and IFA resulted in ~3 log net cell kill, and IPM gave a 2 log net cell kill against B16 melanoma, while PM caused only a marginal tumour delay. Against the ovarian tumour, all but PM gave ~2 log net cell kill. PM gave a 1 log net cell kill against this tumour on a multiple-dose schedule.

IPM, PM, IFA, and CPA showed only limited activity against s.c. growing, advanced-stage (100–400 mg size) colon adenocarcinoma 06/A in CDF1 mice. At the maximum tolerated dosage levels ($LD_{10}$ or less), q7 day (d) × 3 i.v. treatment (CPA-63 mg kg$^{-1}$/dose; PM, 63 mg kg$^{-1}$/dose; IPM, 100 mg kg$^{-1}$/dose; IFA, 160 mg kg$^{-1}$/dose), there were no partial or complete tumour regressions. IPM was, however, at least as active as CPA based on tumour growth delay to 1250 mg (T − C) and increase in life span over that of untreated controls (ILS) (IPM = 14 day T − C, 116% ILS compared with CPA = 6.3 day T − C, 58% ILS).

Evaluation of IPM was performed against a single rat tumour, Yoshida ascitic sarcoma, the signal tumour used to select candidate oxazaphosphorines for further investigations (Arnold *et al.*, 1961). Results are shown in Table VIII and indicate no clear differences in antitumour activity for single-dose treatment with IPM, CPA, IFA or PM or multiple-dose treatment with the latter.

Confirmation of the activity of IPM was obtained in the National Cancer Institute's experimental tumour test panel. IPM passed Decision Network 2

for L1210 and P388 leukaemias, Lewis lung carcinoma, B16 melanoma, colon tumour 38, and mammary tumour CD8F1. IPM was inactive against a lung tumour xenograft and a mammary tumour xenograft.

*Pharmacokinetics and pharmacodynamics of L1210 leukaemia cell killing* In order to determine what range of values of the pharmacokinetic parameter, concentration *times* time (C x T), is efficacious for appreciable L1210 leukaemia cell kill, IPM, 4-hydroxycyclophosphamide (the activated metabolite of CPA), PM, and acrolein, a toxic metabolite of CPA and IFA (Alarcon *et al.*, 1972), were incubated with L1210 cells at 35°C in a shaker water bath at various concentrations for various times. Cell kill *in vitro* was determined by bioassay *in vivo* by implanting aliquots of the cell/drug suspension in female CDF1 mice and observing for life span (Schabel *et al.*, 1977). Several times were compared with those of control mice receiving titrated numbers of cells from a cell suspension incubated without drug, and the results are shown in Table IX. Acrolein was the most cytotoxic agent, causing a 4-

**Table VIII** Effect of isophosphoramide mustard, phosphoramide mustard, cyclophosphamide and ifosfamide against Yoshida sarcoma in rats. Implant $10^7$ cells i.p., 80% take rate; drug treatment day 1; duration of expt. = 120 days

| *Agent* | *Dose ($mg\,kg^{-1}$)* | *Schedule* | *Percent day 120 survivors* | *Percent ILS* (dying animals only)* |
|---|---|---|---|---|
| Isophosphoramide mustard | 100 | Single dose | 60 | 514 |
| | 75 | | 80 | 492 |
| Phosphoramide mustard | 140 | Single dose | 90 | 71 |
| | 105 | | 90 | 0 |
| | 70 | | 90 | 114 |
| | 20 | q5 min x 7 | 90 | 828 |
| | 15 | | 100 | ~ |
| Cyclophosphamide | 225† | Single dose | 20 | 71 |
| | 150 | | 90 | −58 |
| Ifosfamide | 225 | Single dose | 100 | ~ |
| | 150 | | 90 | −58 |

*Median survival time among the 16/20 control animals that dies was 7 days.
†Toxic dose.

**Table IX** L1210 leukaemia cell kill *in vitro* with isophosphoramide mustard phosphoramide mustard, 4-hydroxycyclophosphamide and acrolein

| *Conc. ($\mu g\,ml^{-1}$)* | *Exposure time (min)* | *$Log_{10}$ cell kill* IPM | *PM* | *HOCPA* | *Acrolein* | *$\mu g \cdot min\,ml^{-1}$* |
|---|---|---|---|---|---|---|
| 0.1 | 1 | <1 | <1 | <1 | <1 | |
| 1 | 1 | <1 | <1 | <1 | 1 | |
| 10 | 1 | <1 | <1 | <1 | 4 | |
| 100 | 1 | 3 | 1 | 5 | >7 | $10^2$ |
| 0.1 | 10 | <1 | <1 | <1 | 1 | |
| 1 | 10 | <1 | <1 | 1 | 1 | |
| 10 | 10 | <1 | <1 | 1 | 7 | |
| 100 | 10 | 6 | 3 | 7 | >7 | $10^3$ |
| 0.1 | 100 | <1 | <1 | <1 | <1 | |
| 1 | 100 | <1 | <1 | <1 | <1 | |
| 10 | 100 | 5 | 2 | 6 | >7 | $10^3$ |
| 100 | 100 | >7 | 7 | >7 | >7 | $10^4$ |

Drugs were incubated with $2.5 \times 10^7$ cells/ml in physiological saline at 35°C.

log reduction at a C×T value of only 10 $\mu g \cdot min\, ml^{-1}$. Both 4-hydroxycyclophosphamide and IPM exhibited significant cytotoxicity at 100 $\mu g \cdot min\, ml^{-1}$ at high concentration and short-to-moderate exposure time, and at 1000 $\mu g \cdot min\, ml^{-1}$ at high concentration and moderate exposure time, as well as at moderate concentration and long exposure time.

The duration of therapeutically-effective blood levels of CPA, IFA, PM, and IPM was determined by treating female CDF1 mice with drug (i.p., $LD_{10}$ dose) and implanting various groups with $10^7$ L1210 cells i.p. 10, 20, 30, or 40 min later. The results indicated that IPM (200 $mg\, kg^{-1}$) was capable of killing 6 $log_{10}$ units of cells for 20 min compared with 30 min for IFA (450 $mg\, kg^{-1}$). Comparable effectiveness for PM (210 $mg\, kg^{-1}$) and CPA (300 $mg\, kg^{-1}$) was 25 min and 40 min, respectively.

*Mutagenic activity* IPM and 2-chloroethylamine, a potential hydrolytic decomposition product of both IPM and carboxyifosfamide *in vivo*, were compared in the Ames mutagenicity test (Ames *et al.*, 1975) with PM and bis(2-chloroethyl)amine, products of metabolism or hydrolytic degradation of CPA or its metabolites (Jardine *et al.*, 1978). The results (data not presented) indicate greater mutagenic activity for these alkylating metabolites of CPA [PM and bis(2-chloroethyl)amine] than for those of IFA [IPM and 2-chloroethylamine].

### Discussion

IPM was synthesized in satisfactory yield by reaction of phenyl phosphorodichloridate with 2-chloroethylamine followed by catalytic hydrogenolysis of the phenyl group. Although the synthesis of IPM has not been described previously, it was prepared in 1968 by Brock & Arnold (private communication), and several reports of its chemical and toxicological properties were reported previously (Brock & Hohorst, 1977; Brock *et al.*, 1979; Hohorst, 1977; Rauen & Schriewer, 1971).

Investigation of the metabolism of IFA in experimental animals and in humans has established the metabolic pathway shown in Figure 1. As expected, no major qualitative differences in metabolism of IFA and CPA were observed. The isomeric structure of IFA results in the production of *N*-dechloroethylifosfamide, a metabolite identified by Norpoth *et al.* (1976) in humans, by Takamizawa *et al.* (1974) in rabbits, and by us (Struck, 1976) in mice and dogs.

IPM is an effective agent against several murine leukaemias and solid tumours, including leukaemias L1210 and P388 that are partially resistant to CPA and IFA. As well as PM, it is also active against Yoshida ascitic sarcoma in rats. This is in contrast to results of Brock & Hohorst (1977), who reported low activity for PM but high relative activity for CPA and its 4-hydroxy derivative against this tumour. It was closely comparable in efficacy to CPA and IFA at optimum doses for each of these agents, against every tumour except i.c. implanted P388 leukaemia. The relative inactivity of IPM and PM against the latter, in contrast to the activity demonstrated by IFA and CPA, suggests a possible role for their hydroxylated metabolites as transport forms against this tumour (Brock & Hohorst, 1977; Colvin *et al.*, 1976, Cox *et al.*, 1975, Domeyer & Sladek, 1980). The comparable activity of IPM and CPA against 4 of the tumours described previously was confirmed by the National Cancer Institute, and, in addition, IPM was shown to be active against mouse colon tumour 38.

Conclusive data have not yet been obtained to permit identification of the metabolite(s) of CPA and IFA responsible for the cytotoxic action against tumour cells. Brock & Hohorst (1977) insist that the selectivity for tumour toxicity resides exclusively with 4-hydroxy CPA and 4-hydroxy-IFA (or the isomeric aldophosphamide and aldoifosfamide). Others (Colvin *et al.*, 1976; Cox *et al.*, 1975; Domeyer & Sladek, 1980: Hilton *et al.*, 1981; Sladek, 1977; Sladek *et al.*, 1981; Sladek & Powers, 1980) are totally or partially in agreement with this view and suggest that the hydroxylated metabolites are at least important extracellular determinants of antitumour activity. In contrast, some (Friedman *et al.*, 1976; Struck *et al.*, 1975) have suggested that the total or dominant cytotoxicity is controlled by extracellular PM and IPM, a view consistent with the high, CPA- and IFA-comparable, antitumour activity reported herein.

Because of certain observations on DNA cross-linking (Hilton *et al.*, 1981) and cellular transport (Lenssen & Hohorst, 1979), it could be suggested that the mechanisms of action of extracellular PM and of 4-hydroxycyclophosphamide are different. The superiority of 4-hydroxycyclophosphamide (or its precursor 4-hydroperoxycyclophosphamide) to PM and IPM *in vitro* has been observed in most evaluations (Brock & Hohorst, 1977; Hilton *et al.*, 1981; Sladek & Powers, 1980), including our own (Table VIII), whereas *in vivo* the differences in many evaluations (Friedman *et al.*, 1976; Ramonas *et al.*, 1981) but not all (Brock & Hohorst, 1977) are less pronounced or even disappear, particularly with IPM as shown in the present work. Is the reason for these differences possibly based on pharmacokinetic properties, as our results on duration of optimum cell killing factor(s) intimate; is it a function of cell uptake, or is it that extracellular 4-hydroxycyclophosphamide and PM and IPM are acting by different mechanisms, such as, for example, DNA cross-linking by 4-hydroxycyclophosphamide *vs.* membrane alteration and/or non-DNA alkylation by PM and IPM?

Brock & Hohorst (1977) discount any major contribution to selectivity *in vivo* by selective deactivation by normal cells as proposed primarily by Domeyer & Sladek (1980) and Cox *et al.* (1975) or by pharmacokinetic properties as we have done. Data presented herein (Tables I–VIII) indicate comparable selectivity for tumour toxicity *in vivo* for IPM, CPA, and IFA at comparable host toxicity ($LD_{10}$). The extensive data of Friedman, *et al.* (1976), in which PM was compared with CPA against 25 experimental tumours, demonstrated that PM was of comparable or superior activity against 18 of them; these data, coupled with those included herein, clearly demonstrate that extracellularly-delivered PM and IPM are effective agents, in many cases with anti-tumour activity and selectivity *in vivo* comparable to CPA and IFA and their 4-hydroxy and 4-hydroperoxy derivatives. Because these data (Friedman *et al.*, 1976, and those reported herein) can essentially account for the anti-tumour activity of the parent drugs *in vivo*, it seems reasonable to assume that PM and IPM, delivered to tumour cells by circulation, contribute significantly, if not dominantly or exclusively in some cases, to the observed activity of the parent drugs and the primary metabolites. Their anti-tumour effect *in vivo* suggests that either they participate directly in tumour cytotoxicity as extracellulary-delivered metabolites of their parent drugs, or that they are acting by a mechanism different from that of the parent drugs and primary metabolites.

The mutagenicity of IPM and a potential hydrolytic product, 2-chloroethylamine, was found to be less than that of PM and its hydrolytic product, bis(2-chloroethyl)amine. These results suggest reduced likelihood of mutagenic transformation after treatment with IFA or IPM in comparison with CPA or PM.

Haemorrhagic cystitis frequently results from treatment of patients with CPA and IFA (Morgan *et al.*, 1981; Scheef *et al.*, 1979), and this toxic effect has been attributed to the metabolite acrolein (Brock *et al.*, 1979; Cox, 1979). 4-Hydroxycyclophosphamide and 4-hydroxyifosfamide and the corresponding hydroperoxy derivatives similarly yield acrolein upon decomposition. An advantage of PM and IPM is their inability to generate this toxic metabolite, although various methods have been reported (Morgan *et al.*, 1981; Scheef, *et al.*, 1979) to control acrolein toxicity. Direct instillation of the phosphorodiamidic acids into bladders of experimental animals (Brock *et al.*, 1979) demonstrated the absence of generation of urotoxicity in comparison with acrolein. Acrolein has also been shown to cause denaturation of hepatic microsomal cytochrome P-450 (Marinello *et al.*, 1978) and to be embryotoxic in rabbits (Claussen *et al.*, 1980).

IPM is an active alkylating agent and, like PM (Colvin *et al.*, 1976; Struck *et al.*, 1975), requires no additional activation. As such, variability in activation, which is possible after administration of parent drug, is eliminated. Chemically, IPM appears to be more resistant to hydrolytic deactivation than PM. Brock & Hohorst (1977) and Hohorst (1977 reported a hydrolysis half-life of both 840 min and 2.6 h for IPM, compared to 48 min, 180 min, and 542 min for PM, 4-hydroxycyclo phosphamide, and 4-hydroxyifosfamide, respectively, in 0.07 M phosphate buffer at 37°C.

Precise quantitative data have not yet been obtained for the pharmacokinetics of IPM, but our results demonstrate faster clearance of optimum levels of L1210 cell killing factor(s) than occurs with CPA and IFA. This property could be disadvantageous in that tumour cell exposure to the cytotoxic agent is less than for parent drug, thus possibly requiring a divided-dose schedule for optimum response against some tumours. Faster clearance could also be advantageous in that general systemic exposure to the alkylating agent would be reduced, possibly resulting in less toxicity than that caused by parent drug.

This investigation was supported by Grant Number CA 26632 from the National Cancer Institute, NIH, DHHS.

### References

ALARCON, R.A., MEIENHOFER, J. & ATHERTON, E. (1972). Isophosphamide as a new acrolein-producing antineoplastic isomer of cyclophosphamide. *Cancer Res.*, **32**, 2519.

ALBERTS, D.S., PENG, Y.M., CHEN, H.S. & STRUCK, R.F. (1978). Effect of phenobarbital on plasma levels of cyclophosphamide and its metabolites in the mouse. *Br. J. Cancer*, **38**, 316.

AMES, B.N., McCANN, J. & YAMASAKI, E. (1975). Methods for detecting carcinogens and mutagens with the *Salmonella*/mammalian-microsome mutagenicity test. *Mutat. Res.*, **31**, 347.

ARNOLD, H., BOURSEAUX, F. & BROCK, N. (1958). Neuartige Krebs-Chemotherapeutika aus der Gruppe der zyklischen N-Lost-Phosphamidester. *Naturwissenschaften*, **45**, 64.

ARNOLD, H., BOURSEAUX, F. & BROCK, N. (1961). Über Beziehungen zwischen chemischer Konstitution und cancerotoxicher Wirkung in der Reihe der Phosphamidester des Bis-($\beta$-chlorathy)-amins. *Arnein.-Forsch.*, **11**, 143.

BAKKE, J.E., FEIL, V.J., FJELSTUL, C.E. & THACKER, E.J. (1972). Metabolism of cyclophosphamide by sheep. *J. Agr. Food Chem.*, **20**, 384.

BROCK, N. (1968). Nouveaux esters phosphamides de moutarde azotée et leur activité cytostatique. *Laval Méd.*, **39**, 696.

BROCK, N. & HOHORST, H-J. (1977). The problem of specificity and selectivity of alkylating cytostatics: studies on N-2-chloroethylamido-oxazaphosphorines. *Z. Krebsforsch.*, **88**, 185.

BROCK, N., STEKAR, J., POHL, J., NIEMEYER, U. & SCHEFFLER, G. (1979). Acrolein, the causative factor or urotoxic side-effects of cyclophosphamide, ifosfamide, trofosfamide and sufosfamide. *Arzneim.-Forsch.*, **29**, 659.

BRYANT, B.M., JARMAN, M. FORD, H.T. & SMITH, I.E. (1980). Prevention of isophosphamide-induced urothelial toxicity with 2-mercaptoethanesulphonate sodium (mesnum) in patients with advanced carcinoma. *Lancet*, **ii**, 657.

BURKERT, H. (Ed.) (1977). *International Holoxan$^{R}$-Symposium Proceedings*, Düsseldorf, D-48 Bielefeld 14: Asta-Werke, A.G.

CLAUSSEN, U., HELLMANN, W. & PACHE, G. (1980). The embryotoxicity of the cyclophosphamide metabolite acrolein in rabbits, tested in vivo by i.v. injection and by the yolk-sac method. *Azrneim.-Forsch.*, **30**, 2080.

COLVIN, M., BRUNDRETT, R.B., KAN, M-N.N., JARDINE, I. & FENSELAU, C. (1976). Alkylating properties of phosphoramide mustard. *Cancer Res.*, **36**, 1121.

CORBETT, T.H., GRISWOLD, D.P., Jr., ROBERTS, B.J., PECKHAM, J.C. & SCHABEL, F.M., Jr. (1977). Evaluation of single agents and combinations of chemotherapeutic agents in mouse colon carcinomas. *Cancer*, **40**, 2660.

CORBETT, T.H., GRISWOLD, D.P., Jr., WOLPERT, M.K., VENDITTI, J.M. & SCHABEL, F.M., Jr. (1979). Design and evaluation of combination chemotherapy trials in experimental animal tumour systems. *Cancer Treat. Rep.*, **63**, 799.

COX, P.J. (1979). Cyclophosphamide cystitis–identification of acrolein as the causative agent. *Biochem. Pharmacol.*, **28**, 2045.

COX, P.J., PHILLIPS, B.J. & THOMAS, P. (1975). The enzymatic basis of the selective action of cyclophosphamide. *Cancer Res.*, **35**, 3755.

DOMEYER, B.E. & SLADEK, N.E. (1980). Kinetics of cyclophosphamide biotransformation *in vivo*. *Cancer Res.*, **40**, 174.

FRIEDMAN, O.M., WODINSKY, I. & MYLES, A. (1976). Cyclophosphamide-related phosphoramide mustards—recent advances and historical perspective. *Cancer Treat. Rep.*, **60**, 337.

GERAN, R.I., GREENBERG, N.H., MACDONALD, M.M., SCHUMACHER, A.M. & ABBOTT, B.J. (1972). Protocols for screening chemical agents and natural products against animal tumours and other biological systems (Third Edition). *Cancer Chemother. Rep.* (Part 3) **3**, 2.

HILL, D.L., LASTER, W.R., Jr., KIRK, M.C., EL DAREER, S. & STRUCK, R.F. (1973). Metabolism of iphosphamide and production of a toxic iphosphamide metabolite. *Cancer Res.*, **33**, 1016.

HILTON, J., COHEN, D. & COLVIN, M. (1981). DNA crosslinking in L1210 cells sensitive and resistant to cyclophosphamide. *Proc. Am. Assoc. Cancer Res.*, **22**, 233.

HOHORST, H-J. (1977). The problem of specificity and selectivity of N-(2-chloroethyl)-amido-oxozaphosphorines, in *International Holoxan$^{R}$-Symposium Proceedings*, (Ed. Burkert). D-48 Bielefeld 14: Asta-Werke, A.G.

JARDINE, I., FENSELAU, C., APPLER, M., KAN, M-N., BRUNDRETT, R.B. & COLVIN, M. (1978). Quantitation by gas chromatography-chemical ionization mass spectrometry of cyclophosphamide, phosphoramide mustard, and nornitrogen mustard in the plasma and urine of patients receiving cyclophosphamide therapy. *Cancer Res.*, **38**, 408.

LENSSEN, U. & HOHORST, H-J. (1979). Zur Frage der Permeabilitat von N,N-Bis(2-chloroathyl)-phosphorsaurediamid in Tumorzellen. *J. Cancer Res. Clin. Oncol.*, **93**, 161.

MARINELLO, A., GURTOO, H.L., STRUCK, R. & PAUL, B. (1978). Denaturation of Cytochrome P-450 by cyclophosphamide metabolites. *Biochem. Biophys. Res. Commun.*, **83**, 1347.

MORGAN, L.R., POSEY, L.E., RAINEY, J. & 4 others. (1981). Ifosfamide: a weekly dose fractionated schedule in bronchogenic carcinoma. *Cancer Treat. Rep.*, **65**, 693.

NORPOTH, K. (1976). Studies on the metabolism of isophosphamide in man. *Cancer Treat. Rep.*, **60**, 437.

RAMONAS, L.M., ERICKSON, L.C., KLESSE, W., KOHN, K.W. & ZAHARKO, D.S. (1981). Differential cytotoxicity and DNA crosslinking produced by polymeric and monomeric activated analogues of cyclophosphamide in mouse L1210 leukaemia cells. *Mol. Pharmacol.*, **19**, 331.

RAUEN, H.M. & SCHRIEWER, H. (1971). Alkylans-Alkylandum-Reaktionen. 4-2-Chloräthylamin und Phosphamidverbindungen als Alkylantien. *Arzeim.-Forsch.*, **21**, 518.

SCHABEL, F.M., GRISWOLD, D.P., Jr., LASTER, W.R., Jr., CORBETT, T.H. & LLOYD, H.H. (1977). Quantitative evaluation of anticancer agent activity in experimental animals. *Pharmacol. Ther.* (Part A) **1**, 411.

SCHEEF, W., KLEIN, H.O., BROCK, N., BURKERT, H., GUNTHER, U., HOEFER-JANKER, H., MITRENGA, D., SCHNITKER, J. & VOIGTMANN, R. (1979). Controlled clinical studies with an antidote against the urotoxicity of oxazaphosphorines:preliminary results. *Cancer Treat. Rep.*, **63**, 501.

SLADEK, N.E. (1977). Cytotoxic activity of alkylating agents in the presence of centrophenoxine and its hydrolysis products. *J. Pharmacol. Exp. Ther.*, **203**, 630.

SLADEK, N.E., BORCH, R.F. & LOW, J.E. (1981). Phosphate catalyzed conversion of 4-hydroperoxycyclophosphamide and 4-hydroxycyclophosphamide to phosphoramide mustard and acrolein. *Proc. Am. Assoc. Cancer Res.*, **22**, 214.

SLADEK, N.E. & POWERS, J.F. (1980). Cytotoxic activity and 4-hydroxycyclophosphamide and phosphoramide mustard concentrations in the plasma of cyclophosphamide treated rats. *Pharmacologist,* **22,** 175.

STRUCK, R.F. (1976). A time study of metabolism of isophosphamide. *Abstr. Meeting Am. Chem. Soc.,* 172nd, Med:44.

STRUCK, R.F., KIRK, M.C., WITT, M.H. & LASTER, W.R., Jr. (1975). Isolation and mass spectral identification of blood metabolites of cyclophosphamide: evidence for phosphoramide mustard as the biologically active metabolite. *Biomed. Mass. Spectr.,* **2,** 46.

TAKAMIZAWA, A., MATSUMOTO, S., IWATA, T. & 4 others. (1974). Synthesis and metabolic behavior of the suggested active species of isophosphamide having cytostatic activity. *J. Med. Chem.,* **17,** 1237.